# Awareness of chronic disease related health benefits of physical activity among residents of a rural South Indian region: a cross-sectional study

**DOI:** 10.1186/1479-5868-11-27

**Published:** 2014-02-27

**Authors:** Sundar Kumar Veluswamy, Arun G Maiya, Suma Nair, Vasudeva Guddattu, Narayanapillai Sreekumaran Nair, Sudha Vidyasagar

**Affiliations:** 1 Department of Physiotherapy, School of Allied Health Sciences, Manipal University, Manipal, India; 2 Dr. TMA Pai Endowment Chair in Exercise Science and Health Promotion, Manipal University, Manipal, India; 3 Department of Community Medicine, Kasturba Medical College, Manipal University, Manipal, India; 4 Department of Statistics, Manipal University, Manipal, India; 5 Department of Medicine, Kasturba Medical College, Manipal University, Manipal, India

**Keywords:** Perceived health benefits, Knowledge, Lower-middle income countries, Leisure time physical activity

## Abstract

**Background:**

Physical activity trends for a lower-middle income country like India suggest a gradual decline in work related physical activity and no concomitant increase in leisure time physical activity. Perceived health benefits of physical activity and intention to increase physical activity have been established as independent correlates of physical activity status. In India, not much is known about peoples’ perceptions of health benefits of physical activity and their intention to increase physical activity levels. This study was performed to understand peoples’ perceptions and awareness about health benefits of physical activity in a rural South Indian region.

**Methods:**

This cross-sectional study was conducted using a multistage cluster sampling design. A content validated, field tested questionnaire was administered in person by a trained interviewer in the participants’ native language. The questionnaire assessed the participants’ perceptions about their lifestyle (active or sedentary), health benefits of physical activity and need for increasing their physical activity. In addition, the participant’s physical activity was assessed using version 2 of global physical activity questionnaire. Frequencies and percentages were used to summarise perceived health benefits of physical activity and other categorical variables. Age and body mass index were summarised using mean ± SD, whereas physical activity (MET.min.wk ^−1^) was summarised using median and interquartile range.

**Results:**

Four hundred fifty members from 125 randomly selected households were included in the study, of which 409 members participated. 89% (364) of participants felt they lead an active lifestyle and 83.1% (340) of participants did not feel a need to increase their physical activity level. 86.1%, (352) of the participants were physically active. Though 92.4% (378) of participants felt there were health benefits of physical activity, majority of them (75.1%) did not report any benefit related to chronic diseases. None mentioned health benefits related to heart disease or stroke.

**Conclusion:**

There is low awareness of chronic disease related benefits of physical activity and participants do not see a need to increase their physical activity level. Public health awareness programs on importance and health benefits of physical activity would be useful to counter the anticipated decline in physical activity.

## Background

Physical inactivity is a key independent modifiable risk factor for chronic diseases and all-cause mortality [1]. As the fourth leading risk factor for global mortality, physical inactivity accounted for an estimated 3.2 million deaths in 2004 [2]. Among the three domains of physical activity (PA) viz., work (comprising of both occupational and domestic PA), travel and leisure time, PA trends suggest an overall decline in work related PA [3]. With the burden of chronic diseases reaching epidemic proportions in a lower-middle income country (LMIC) like India, this trend assumes greater significance where leisure time physical activity (LTPA) levels are significantly low [4-8]. In most LMICs including India, people generally adopt PA as part of their work and travel needs rather than as part of leisure time activity [9]. Among the five countries (USA, UK, China, Brazil and India) for which PA projections for 2030 are available, India does not demonstrate a positive trend in LTPA as is seen in other four countries [10]. In addition, modernization and a gradual shift towards a sedentary lifestyle will inevitably result in a progressive decline in work related PA. The negative health impact of decline in work related PA on chronic disease burden could be countered by a greater increase in LTPA.

Promoting adoption and maintenance of LTPA is a major challenge as it is influenced by multitude of factors like demographic and biological factors; psychological, cognitive and emotional factors; behavioural attributes and skills; social and cultural factors; physical environmental factors and physical activity characteristics [11]. Theoretical models like Health belief model (HBM), Social cognitive theory and Transtheoretical model (TTM) have been used to understand physical activity behaviour [12]. The US Department of Health and Human Services, in their report on PA, identified self confidence in ones’ ability to be active and having a positive opinion about the benefits of PA as important factors influencing PA participation [13]. The report encourages using behavioural change theories like HBM and TTM in understanding and designing interventions for PA promotion. Both HBM and TTM place a greater emphasis on an individual’s perception about his health and corrective action. HBM hypothesizes that an individual’s likelihood of undertaking health behaviour (for example, become physically active) depends on one’s perception of his susceptibility to a particular illness (risk of developing coronary artery disease), seriousness of the consequence if he acquires the health problem (need for surgery, cost of treatment, loss of work etc.) and the belief in the health behaviours’ ability to reduce the threat of acquiring the illness (effect of PA in reducing the risk of coronary artery disease) [14-16]. On the other hand, TTM postulates that an individual attempting to change towards healthy behaviour moves through a series of stages viz., pre-contemplation, contemplation, preparation, action and maintenance. An individual at the ‘Pre-contemplation stage’ is not aware of his health risks and the need to change his behaviour whereas the individual in the ‘Contemplation stage’ is aware of the risks, understands the benefits of healthy behaviour and is contemplating taking action. An individual is said to be in ‘Preparation stage’ if he is seen to be taking small changes in behaviour but still not meeting the criteria for changed behaviour. An individual is considered to be in the ‘Action stage’ if he meets the criteria for the changed behaviour for duration of less than six months and in the ‘Maintenance stage’ if he meets the criteria of changed behaviour for greater than six months. The stages are not linear and individuals move back and forth between stages in a spiral manner [17,18]. In essence, an individual who is not aware of his health risk and does not understand the benefits of health behaviour is unlikely to be prepared to change his behaviour.

Studies based on these models have found that knowledge and positive beliefs about benefits of PA have a greater role in predicting PA adoption [19,20]. Using theoretical constructs of these models, it could be inferred that an individual who is aware of the risks of developing chronic diseases and the role of PA in reducing them is more likely to adopt PA than an individual who is not aware of the benefits of physical activity.

This study was undertaken in a rural coastal South Indian region that is known to have high prevalence of chronic disease risk factors [21-25]. Not much is known about PA levels of this region and PA perceptions of its residents. This study aimed to understand the awareness of health benefits of PA, perception about current PA and PA level of this population. Against the backdrop of decreasing work related physical activity, understanding peoples’ attitude towards PA and awareness of its health benefits based on the constructs of HBM and TTM will enable us understand the strategies needed to promote PA. This information could help policy makers initiate appropriate PA intervention programs and provide insights to other LMICs like India that are researching measures to promote PA.

## Methods

### Design and setting

**Figure 1:**
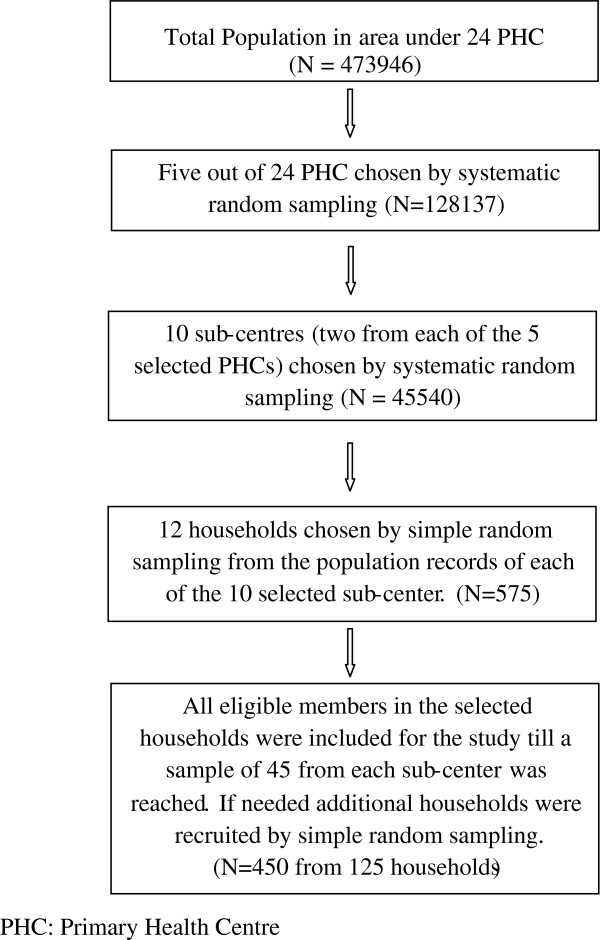
Flowchart of sampling procedure and participant recruitment.

This cross-sectional study was conducted in a rural coastal taluk in the Indian state of Karnataka as part of a larger study aimed to create a PA profile of the region. A multistage cluster sampling design was adopted. As per the 2011 district health and family welfare office records, the rural taluk under study had a population of 473,946. This taluk had 24 primary health centres (PHC) that catered to the healthcare needs of the area. The PHCs were taken as the primary sampling units. Each PHC catered to between two to nine sub-centres which served as secondary sampling units. Households were randomly selected from each of the selected sub-centres, the residents of which served as the final units. Details of the sampling procedure are described in Figure 1.

The sample size was estimated (considering a PA prevalence of 50% [based on pilot study data]; with a relative precision of 15% at 95% level of confidence; a design effect of two and 20% non-respondent rate) as 428 and was rounded off to 450. Based on this number, it was decided to sample 45 individuals from each of the 10 selected sub-centres.

### Participants and measurement

The study was conducted using a content validated, field tested questionnaire administered in person by a single interviewer in the participant’s native language. The questionnaire had a combination of both open and closed ended questions that assessed the participants’ perceptions about their lifestyle (active or sedentary), health benefits of PA and their perceived need for increasing PA levels for health benefits. Active lifestyle was defined as participating in at least 30 minutes of moderate intensity PA per day on most days of the week [26-28]. Sedentary lifestyle was defined as a level of PA that is less than 30 minutes of moderate intensity physical activity on most days of the week [27-30]. Participants who felt there are health benefits of PA were asked to list the perceived benefits. Participants who perceived a need to increase their PA were asked if they attempted to increase their PA in the past six months. In addition, the participant’s total PA and PA in work, travel and leisure time domain was assessed using version 2 of global physical activity questionnaire [31-34]. Based on their total moderate to vigorous PA in MET.min.wk^−1^, participants were classified as physically active for PA ≥ 600 MET.min.wk^−1^ and as physically inactive for PA < 600 MET.min.wk^−1^. In addition, information on the participants’ age, highest education and employment status were obtained during the interview. Body mass index (BMI) was calculated from height (in meters) and weight (in kilograms) measurements performed using portable stadiometer and weighing scale.

A local health worker introduced the interviewer to the head of the selected household who provided a list of members of the family. All adult members in the age range of 18 – 64 years and living in the selected household for greater than six months were included in the study. Members not consenting to participate in the study or who were not available for the interview after five visits were considered as non-responders. The study protocol was approved by the institutional ethical committee, Kasturba Hospital, Manipal and all participants provided written informed consent.

### Data analysis

Frequencies and percentages were used to summarise perceived health benefits of PA and other categorical variables. Age and BMI were summarised using mean ± SD, whereas PA (MET.min.wk^−1^), was summarised using median and interquartile range. Generalised estimating equations (GEE) with exchangeable correlational structure for members within a household was used to assess the association of PA status with perceived lifestyle and the need to increase PA. A p value ≤ 0.05 was considered to be statistically significant. All analysis was carried out using SPSS v15, Bangalore, South Asia.

## Results

A total of 575 adult residents living in 125 randomly selected households were screened for inclusion in the study, of which, 450 members met the criteria. The study had a response rate of 90.9% (409/450). Demographic details of the participants are summarised in Table 1. Only 13.2% (54/409) of participants had completed graduation or more. The most common occupational categories were home maker (%, n: 32.3%, 132), unskilled labour (20.5%, 84), student (6.1%, 25), agriculture (5.9%, 24), clerical (5.9%, 24), skilled technician (3.9%, 16) and driver (3.9%, 16). Majority of the participants were physically active (86.1%, 352/409). Among the domains of PA, 80% (327/409) reported participating in work related PA, whereas 64.3% (263/409) of them reported participating in travel related PA and only 18.6% (76/409) of them participated in LTPA.

**Table 1:** Demographic characteristics of participants

**Characteristics**	**Males (N = 189)**	**Females (N = 220)**	**Total (N = 409)**
**Age** (years), mean ± SD	39.1 ± 13.6	37.7 ± 12.1	38.4 ± 12.8
**BMI** (kgm^−2^), mean ± SD	21.3 ± 4.3	21.1 ± 4.6	21.2 ± 4.5
**Education, n (%)**			
Primary or Less,	86 (45.5)	118 (53.6)	204 (49.9)
More than primary but less than graduation	80 (42.3)	71 (32.3)	151 (36.9)
Graduation or more	23 (12.2)	31 (14.1)	54 (13.2)
*PA in various domains, median (IQR)			
Work	1680 (0, 8400)	2460 (960, 3360)	1920 (420, 5040)
Travel	560 (0, 1080)	360 (0, 840)	480 (0, 840)
**Leisure	0 (0, 480)	0 (0, 0)	0 (0, 0)
Total PA	3720 (1440, 10080)	2910 (1680, 4540)	3120 (1680, 6720)
***PA Category, n (%)			
Active	157 (83.1)	195 (88.6)	352 (86.1)
Inactive	32 (16.9)	25 (11.4)	57 (13.9)

BMI – Body Mass Index; PA – Physical Activity; IQR – Interquartile range;

MET – Metabolic Equivalent of Task.

*PA in MET.min wk^−1^.

**LTPA was very low resulting in zero as median and IQ range.

***Participants were categorised as Inactive for PA < 600 MET.min.wk^−1^ and as active for PA ≥ 600 MET.min.wk^−1^.

The participants’ perceptions about their lifestyle, benefits of PA, need to increase their PA for health benefits and attempts to increase PA are summarised in Table 2. Among the 43 participants who wanted to increase their PA for health benefits, 39.5% (17/43) wanted to increase it for improving health, 30.2% (13/43) for increasing strength and 25.6% (11/43) for reducing or controlling weight.

**Table 2:** Participants' perceptions of their lifestyle, health benefits of PA, need and attempt to increase their PA for health benefits

	**Males (N = 189)**	**Females (N = 220)**	**Total (N = 409)**
**Perceived lifestyle, n (%)**			
Active	164 (86.8)	200 (90.9)	364 (89)
Sedentary	25 (13.2)	20 (09.1)	45 (11)
**Health benefits of PA, n (%)**			
Yes	174 (92.1)	204 (92.7)	378 (92.4)
No	7 (03.7)	6 (02.7)	13 (03.2)
Don’t know	8 (4.2)	10 (4.5)	18 (4.4)
**Need to increase their PA for health benefits, n (%)**			
Yes	26 (13.7)	17 (7.7)	43 (10.5)
No	153 (81.0)	187 (85)	340 (83.1)
Don’t know	10 (5.3)	16 (07.3)	26 (06.4)
*Attempted to increase PA in the previous six months (n = 43; males 26, females 17), n (%)			
Yes	13 (50)	4 (23.5)	17 (39.5)
No	13 (50)	13 (76.5)	26 (60.5)

PA – Physical Activity.

*Answered by only those who felt a need to increase their PA for health benefits.

Associations between PA status (active or inactive) with perceived lifestyle (active or sedentary) and perceived need to increase PA (No or Yes) is highlighted in Table 3. Participants who perceived leading an active lifestyle were 10.5 (95% CI: 5.2, 21.1; p < 0.001) times more likely to be active than those who felt they have a sedentary lifestyle. Participants who did not perceive a need to increase their activity level were 1.8 (95% CI: 0.7, 4.9; p = 0.198) times likely to be active than those who felt they need to increase their physical activity.

**Table 3:** Association of PA status with perceived lifestyle and need to increase PA

	**Physical activity status (N = 409)**		**Wald chi-square (df), p value**
	**Physically active (n = 352)**	**Physically inactive (n = 57)**	
**Perceived lifestyle**			
Active (n = 364)	331 (90.9%)	33 (9.1%)	43.971 (1), p < 0.001
Sedentary (n = 45)	21 (46.7%)	24 (53.3%)	
**Perceived need to increase PA**			
No (n = 366)*	322 (88%)	44 (12%)	1.659 (1), p = 0.198
Yes (n = 43)	30 (69.8%)	13 (30.2%)	

Note: The cluster effect (household) was adjusted using generalised estimating equations with exchangeable co-relational structure for members within a household and the ICC was estimated to be 0.032.

*For the purpose of analysis, all participants who did not answer ‘Yes’ to the question were considered as not perceiving a need to increase PA.

Participants (n = 378), who felt there are health benefits of PA, were asked to list the benefits perceived by them. The reported benefits were broadly categorised into general benefits and chronic disease related benefits. The reported benefits along with their frequency under both the categories are summarised in Table 4. Majority of the participants (75.1%, 284/378) did not report even one chronic disease related health benefit of PA, while 19.6% (74/378) reported only one benefit and 5.3% (20/378) reported two or more benefits. None mentioned health benefits related to heart disease or stroke.

**Table 4:** Perceived health benefits of physical activity (PA) and their frequencies

**General benefits of PA participation (N = 378)**	Frequency, n (%)*	**Chronic disease related benefits of PA participation (N = 378)**	Frequency, n (%)*
Increases alertness/activeness	129 (34.1)	Reduces/controls weight	46 (12.2)
Helps in relaxation	79 (20.9)	Prevents diseases	37 (9.9)
Reduces stress	60 (15.9)	Reduces/controls BP	11 (2.9)
Increases strength/fitness	59 (15.6)	Reduces/controls cholesterol	11 (2.9)
Promotes sleep	41 (10.8)	Increases life span	8 (2.1)
Improves digestion	38 (10.1)	Prevents/controls diabetes	5 (1.3)
Makes one feel good/happy	32 (8.5)		
Improves flexibility/reduces stiffness	26 (6.9)		
Reduces joint pain	25(6.6)		
Improves appetite/hunger	14 (3.7)		
Improves circulation	13 (3.4)		
Increases endurance	12 (3.2)		
Removes impurities	5 (1.3)		
Increases immunity	3 (0.8)		
Reduces/prevents constipation	3 (0.8)		

*Percentage of responses do not add up to 100% due to non-mutually exclusive responses.

## Discussion

The findings of this study highlight the following: (a) Majority of the participants are physically active; (b) They obtain their physical activity primarily from work and transportation domains with very little participation in LTPA; (c) Most participants did not perceive a need to increase their physical activity level and (d) There is very low awareness regarding chronic disease related health benefits of physical activity.

This pattern of high work related PA and low LTPA is similar to that observed in India’s integrated disease surveillance project in which the mean time spent in work related PA ranged between 128 – 293 minutes per day and that in LTPA ranged between 4 – 67 minutes per day [8]. This trend of low LTPA and greater work related PA is common among most LMICs [9]. The cause of concern here is the anticipated decline in work and transportation related PA. Greater participation in work and transportation related PA in this population is likely to be due to factors other than an objective to accrue health benefits. Though current total PA levels are good in this population, there is a stark possibility of it declining due to economic and social development. A review of best practice in interventions to promote PA in developing countries by World Health Organization summarised that urbanization and economic development, in part, drive the increase in burden of chronic diseases and rise in sedentary behaviour in developing countries [35]. The human development index of the district, of which the taluk under study is a part, is better than most other districts in the state of Karnataka and is higher than the national average [36-38]. In addition, an increase in income and purchasing power, increase in demand for household appliances and automobile like small cars and two-wheeler bikes has been well documented in this district [39]. Increase in household appliances and automobiles would potentially translate to reduction in work and travel related PA with time. As and when this happens, there will be a greater pressure to increase LTPA. But participation in LTPA is predominantly volitional and factors like perceived health benefits of PA and intention to increase PA are likely to have an influence [40,41].

Though it may be possible to infer that participants would not have perceived a need to increase their PA simply because they had high levels of PA, the results indicate that the association between physical activity status and need to increase PA was not significant. A sub-analysis of physically inactive participants (n = 57) highlights that the majority (77.2%, 44/57) did not perceive the need to increase their physical activity. The constructs of HBM and TTM could possibly help us understand this perceived behaviour. The results from this study indicate very low awareness of chronic disease related benefits of PA in this population and are in stark contrast to the results from other countries. In Canada, more than 75% of population across various strata strongly believed that PA helps to prevent heart diseases [42]. Another review highlighted that about 95% of the UK population know the relationship between physical activity and health [43]. Lack of awareness of important health benefits of PA in this study population could possibly explain their reluctance in wanting to increase their PA [19,20].

In the absence of published data, it is not clear if there is a general lack of awareness about chronic disease risk factors or if this low awareness is specific to relationship between PA and chronic diseases. However information from an on-going study and clinical and field experiences from our institute does indicate that there is awareness about the higher burden of heart disease, diabetes and hypertension in this region and factors like smoking, obesity, cholesterol and higher fat intake are contributory to these diseases.

Carefully designed interventions to improve knowledge and positive beliefs about chronic disease related benefits of PA may have the potential to encourage PA adoption. Many countries have put in place structured public health campaigns to educate and improve awareness among the people about the health benefits of PA, its role in prevention of chronic diseases and national PA guidelines [35,44-46]. PA interventions in developing countries have included strategies to raise awareness of importance and benefits of PA. Such intervention programs in many countries have been successful in raising awareness of the importance and benefits of PA. A few countries have even demonstrated an actual increase in PA levels of large proportion of the population [35]. The current study highlights the need for such awareness and intervention programs in regions similar to this to counter the anticipated decline in PA and raising burden of chronic diseases.

In a population where majority are physically active, it will be a challenge to determine factors facilitating voluntary PA participation. Though many correlates of PA have been identified in western population, it is likely that the correlates are different in LMICs. As LMICs such as India are transitioning towards economic development, it is imperative to determine factors influencing PA adoption and participation. Many studies from different LMIC settings are needed to understand factors influencing PA. The role of knowledge and awareness, personal beliefs and cultural influences, gender, education, occupation, socioeconomic status, chronic disease risk status, environmental factors, social support, policies and the like on PA behaviour in LMICs needs to be studied to device effective strategies to arrest declining PA levels.

### Strengths & limitations

The results of this study could be considered representative of this region due to its sampling methodology. Content validation and field testing of the questionnaire prior to the study, use of a single interviewer and training of the interviewer in the local language was done to reduce bias in administering the questionnaire. The support of the local health workers in introducing the interviewer to the participants’ family ensured familiarity and better cooperation from the participants. A few studies in the past have assessed awareness as part of intervention program but to our knowledge, there are few studies from LMICs like India that has addressed the problem of awareness of benefits of PA. Most studies on awareness of benefits of PA have used closed ended questions or a Likert-type scale. Though such a methodology has its own advantages, we felt the use of such a method could bias the participant towards positive response. Open ended question, as used in this study, on the other hand could capture the actual perception of benefit of PA among participants. Despite the strengths of this study, it has several limitations and it should be considered while interpreting its results. The PA status was assessed by a questionnaire. Though GPAQ is being used in large epidemiological studies in developing countries, like any PA questionnaire, it has inherent errors in quantifying PA. An objective measure would have been ideal but could not be used due to funding limitations. As the interview was conducted by a single investigator, time related factors could have affected the PA levels. Care was taken to avoid monsoon and summer months for this purpose. Moreover, as no active intervention targeting awareness was in progress, it is unlikely that the participants’ perceptions would have changed. Even though, use of open ended question helped in obtaining the actual perceptions of people, it limited the possibility of further analysis. Though correlation of participants PA level and awareness of benefit with other factors like their current chronic disease risk profile, educational and socioeconomic status would have added a wealth of information, such measurements were not within the scope of this study. This study was conducted in a rural population and it is not known if the same trend would be observed in the urban population.

## Conclusion

This study highlights low awareness of chronic disease related benefits of PA and the participants’ reluctance to increase their PA level in a population that has high prevalence of chronic disease risk factors. Public health awareness programs on importance and health benefits of PA are needed to help people in regions like these to adopt and increase their PA. This is essential to counter the raising burden of chronic diseases and inevitable reduction in work and travel related PA.

## Abbreviations

BMI: Body mass index; HBM: Health belief model; LMIC: Lower-middle income countries; LTPA: Leisure time physical activity; PA: Physical activity; PHC: Primary health centre; TTM: Transtheoretical model.

## Competing interests

We declare that a part of the results of this study was presented as a poster during National Cardiology Update in Manipal, Karnataka, India (November 2012). We declare no other competing interest.

## Authors’ contributions

SKV contributed towards study conception, design, data collection, data analysis and wrote the first draft of the manuscript. AGM, SN and SV contributed towards study conception, design and manuscript preparation. VG and NSN contributed towards study conception, design, data analysis and manuscript preparation. All authors critically reviewed and approved the final manuscript for submission.
